# Green Flame-Retardant Blend Used to Improve the Antiflame Properties of Polypropylene

**DOI:** 10.3390/polym16101317

**Published:** 2024-05-08

**Authors:** Christian J. Cabello-Alvarado, Marlene Andrade-Guel, Marissa Pérez-Alvarez, Gregorio Cadenas-Pliego, Pascual Bartolo-Pérez, Diego Martínez-Carrillo, Zoe V. Quiñones-Jurado

**Affiliations:** 1 Centro de Investigación en Química Aplicada, Saltillo 25294, Coahuila, Mexico; christian.cabello@ciqa.edu.mx (C.J.C.-A.); marissa.perez@ciqa.edu.mx (M.P.-A.); 2 CONAHCYT—Centro de Investigación en Química Aplicada, Saltillo 25294, Coahuila, Mexico; 3 Centro de Investigación y de Estudios Avanzados del IPN-Unidad Mérida, Departamento de Física Aplicada, Mérida 97310, Yucatán, Mexico; pascual.bartolo@cinvestav.mx; 4 Centro de Investigación en Geociencias Aplicadas, Universidad Autónoma de Coahuila, Nueva Rosita 26830, Coahuila, Mexico; diegomartinez@uadec.edu.mx; 5 Facultad de Ciencias Químicas, Universidad Juárez del Estado de Durango, Durango 34120, Durango, Mexico; zoevineth@gmail.com

**Keywords:** flame-retardant, clinoptilolite, isotactic polypropylene, lignin, melt-extrusion

## Abstract

The flammability properties of polymers and polymeric composites play an important role in ensuring the safety of humans and the environment; moreover, flame-retardant materials ensure a greater number of applications. In the present study, we report the obtaining of polypropylene (PP) composites contain a mixture of two green flame retardants, lignin and clinoptilolite, by melt extrusion. These additives are abundantly found in nature. Fourier transform infrared (FT-IR), thermogravimetric analysis (TGA), mechanical properties, scanning electron microscopy–energy dispersive X-ray spectroscopy (SEM-EDS), cone calorimetry, UL-94, and carbonized residues analysis were carried out. TGA analysis shows that PPGFR-10 and PPGFR-20 compounds presented better thermal stability with respect to PP without flame retardants. The conical calorimetric evaluation of the composites showed that PPGFR-10 and PPGFR-20 presented decreases in peak heat release rates (HRRs) of 9.75% and 11.88%, respectively. The flammability of the composites was evaluated with the UL-94 standard, and only the PPGFR-20 composite presented the V-0 and 5VB classification, which indicates good flame-retardant properties. Additives in the polymer matrix showed good dispersion with few agglomerates. The PPGFR-20 composite showed an FRI value of 1.15, higher percentage of carbonized residues, and UL-94 V-0 and 5VB rating, suggesting some kind of synergy between lignin and clinoptilolite, but only at high flame-retardant concentrations.

## 1. Introduction

Polymers are one of the most widely used materials in everyday life, which present a high flammability that generates toxic gases and smoke during their combustion. Synthetic polymers are generally more flammable compared to natural polymers; for example, polyolefins have heating values comparable to petroleum [1]. Modulating the flammability properties of polymers and polymeric compounds is a relevant objective in materials science because they are directly related to the safety of people and the environment [2,3].

The enormous need for new flame retardants, which are environmentally friendly and halogen-free, has led to the design of bio-based materials and inorganic fillers [4,5,6,7]. The use of bio-based materials is very attractive due to their low price, wide availability, and easy access. Among these natural products, chitosan, lignin, cyclodextrin, and starch stand out. In addition, it should be considered that some non-bio-based materials, such as calcium carbonate, have caused many difficulties, since when used they affect the end-of-life product, causing byproducts [8]. The most common inorganic fillers are metal hydroxides, silicone dioxide, and metal complexes, among others.

Bio-based flame retardants (BFR) are widely used in synthetic polymers, such as polypropylene (PP), polyethylene (PE), polyamides (PAM), polyvinyl chloride (PVC), epoxy resin (ER), ethylene vinyl acetate copolymer (EVA), and polyurethane (PU), among others. The use of BFR in natural polymers is lower, and applications in polylactic acid, cotton, and Jute fabric are reported [4]. 

PP is one of the most used plastics in the world because it has many attractive advantages such as high rigidity and crystallinity, easy processing, low price, heat resistance, and corrosion resistance, in addition to low water absorption and a lightweight dimensional stability [9,10,11,12,13,14]. In the last decade, global applications of PP have increased, particularly in the form of composites. In the Asia-Pacific region, the PP market is forecast to reach 91.98 million tons in 2024. It is expected to reach 121.81 million tons in 2029, with a compound annual growth rate (CAGR) of 5.78% during the forecast period (2024–2029) [15].

Flame-retardant additives are known for their ability to form char during thermal degradation, which decreases the combustion rate of polymeric materials by preventing oxygen from easily reaching the combustion zone. According to the literature, lignin can be modified to increase its flame-retardant properties. One report notes that lignin contains different reactive chemical sites, allowing five types of chemical modification with (1) phosphorus or nitrogen, (2) phosphorus–nitrogen compounds, (3) combination with silica-containing flame retardant, (4) nanoadditives, and (5) metal ions [16]. Most lignins have excellent thermal stability and a high carbon formation capacity; their thermal degradation occurs in the range of 200–600 °C. When the degradation takes place in an inert nitrogen atmosphere at 900 °C, the formation of carbon structures reaches yields of 35–38%, favored by the aromatic chemical structure of lignin [16,17]. Lignin thermally degrades several degrees earlier than PP, so in the degradation of PP/lignin composites, the formation of hydrocarbon radicals take place in the presence of carbonized lignin, which can form a layer that reduces the combustion rate of PP [17]. Lignin is composed of three basic units: coniferyl alcohol, sinapyl alcohol, and p-coumaric alcohol, which are interconnected by bonds with phenolic hydroxyl, methoxyl, and carboxyl groups [18]. Lignin studies have been performed with other flame retardants such as ammonium polyphosphate, ammonium dihydrogen phosphate, and boric acid, and it was found that the lignin obtained from manchurian ash presents good char-forming ability itself compared to the flame retardant evaluated [19]. G. Gallina et al. investigated PP based on lignin and triglycidylisocyanurate (TGC) and other compounds such as monoammonium phosphate (MAP), melamine, and boric acid to be used as flame retardants [20], observing an unexpected decrease in the ignition time of additive PP in relation to pure PP, suggesting that the initial combustion of the flame retardants was before they could play their role in the material. According to the results, PP/TGC + MAP blend had 21% char at 400 °C, resulting in being the best candidate for flame-retardant composite. In other research, De Chirico et al. [17] studied flame retardants for PP based on lignin, observing that the lignin could be a candidate as a flame retardant and also that it increased with small amounts of other known flame-retardant compounds, as well as the fact that the lignin addition did not reduce the elastic modulus [21]. Another study demonstrates the synergy of zeolite with PP systems and halogen-free intumescent flame retardants, as a large reduction in combustion parameters was achieved and a more continuous and compact char layer was formed [22]. Another method used by Iyer et al. where they incorporated the lignin into LDPE or PP to obtain sustainable green hybrids was by solid-state shear pulverization (SSSP), indicating that this method is better than the melt-mixing method because it leads to better lignin dispersion, as well as improving the mechanical properties [23]. In our previous work, the PP/TiO_2_/lignin composite manufactured by melt-extrusion was reported, where the two additives were incorporated at different concentrations, and the synergy of both additives was observed without using a compatibilizer or chemical modification [24].

In this new study, the aim was to improve the flame-retardant properties of polypropylene (PP), in an environmentally friendly way, by adding additives considered green such as zeolite and lignin. Innovating the combination of two green additives such as lignin and clinoptilolite in PP, there are no reports where this combination exists, as the research carried out used these same additives separately. The preparation method was by melt mixing extrusion in one step; none of the additives were chemically modified beforehand, as reported in other studies, in order to reduce process costs at industrial level and to offer a simple and environmentally friendly method.

## 2. Materials and Methods

### 2.1. Materials

Lignin is an alkali powder that was purchased from Sigma Aldrich (St. Louis, MO, USA), and natural zeolite (95% wt. clinoptilolite) was provided by Zeomex S. A. (San Luis Potosi, Mexico). Polypropylene (PP, 0.76 g/10 min. fluid index) was supplied by Polímeros Nacionales (Ciudad de México, Mexico).

### 2.2. Composite Preparation by the Melt Extrusion Process

**Figure 1:**
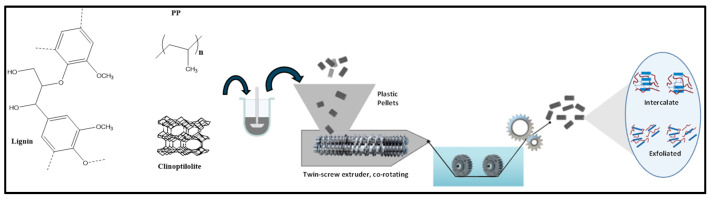
Schematic illustration of the methodology used to obtain polymeric composites by melt-extrusion process.

PP composites with a clinoptilolite/lignin mixture were prepared by melt-mixing extrusion, using a Thermo Scientific Model Prism TSE-24MC laboratory size twin-screw extruder with a screw diameter of 24 mm, an L/D ratio of 40:1, a temperature profile plane of 210 °C, and a rotational speed of 100 rpm. The formulations prepared with the mixture of different amounts of flame retardants and polypropylene are shown in Table 1. For this study, 300 g of sample was prepared; however, the extruder used had a maximum capacity of 3 or 4 Kg per hour depending on the conditions of use and the material processed. Figure 1 schematically illustrates the methodology used in this research, showing how the polymeric composites were obtained through the melt-extrusion process.

### 2.3. Characterization of Composites

#### 2.3.1. Fourier Transform Infrared (FTIR)

Analysis was performed with a Magna Nicolet 550 spectrometer using 100 scans and a resolution of 16 cm^−1^ in the range of 4000–400 cm^−1^.

#### 2.3.2. Thermogravimetric Analysis (TGA)

The thermal stability of the polypropylene and PPGFR composites was evaluated using a TGA Q500 (TA Instruments, New Castle, DE, USA) thermal analyzer. Each specimen was heated from 30 to 600 °C under an N_2_ atmosphere with a flow rate of 50 mL/min and a heating rate of 10 °C/min. After 600 °C, the nitrogen atmosphere was changed to an oxygen atmosphere to accelerate the thermal decomposition of the organic part.

#### 2.3.3. Scanning Electron Microscopy (SEM)

An SEM study was performed directly on the external and cryofractured surfaces of the composites. SEM micrographs were obtained using a JOEL model JSM-7401F field emission scanning electron microscope to determine the dispersion and morphology of the composites.

#### 2.3.4. Cone Calorimetric

Cone calorimeter tests of samples were determined using a Fire Testing Technology Limited (FTT) cone calorimeter, in accordance with ASTM E1354 procedure [25]. The samples had a dimension of 100 mm^2^ and were wrapped in aluminum foil. The FTT provided external heat flux at 35 kW/m^2^ horizontally. Each specimen was analyzed twice, and the average result was obtained. The flame retardancy index (FRI) is represented in Equation (1), and it is defined as the ratio between the pure polymer and the corresponding thermoplastic composite with the flame-retardant additive.

(1) $$Flame\;Retardancy\;Index\;(FRI)=\frac{\left[THR\times \frac{pHRR}{TTI}\right]Neat\;polymer}{\left[THR\times \frac{pHRR}{TTI}\right]Composite}$$

where THR is the total heat release, pHRR is the peak of heat release rate, and TTI is the time to ignition.

The UL-94 vertical burning level of the sample was tested according to ASTM D3801. For the vertical test, five specimens were prepared for each group, and the sample size was 127 mm × 13 mm × 2 mm. Based on the UL-94 standard, the flammability of plastics improves in the following order: HB, V-2, V-1, V-0, 5VB, and 5VA [26].

## 3. Results and Discussion

### 3.1. Chemical Structure Characterization

#### 3.1.1. Fourier Transform Infrared FTIR (ATR)

**Figure 2:**
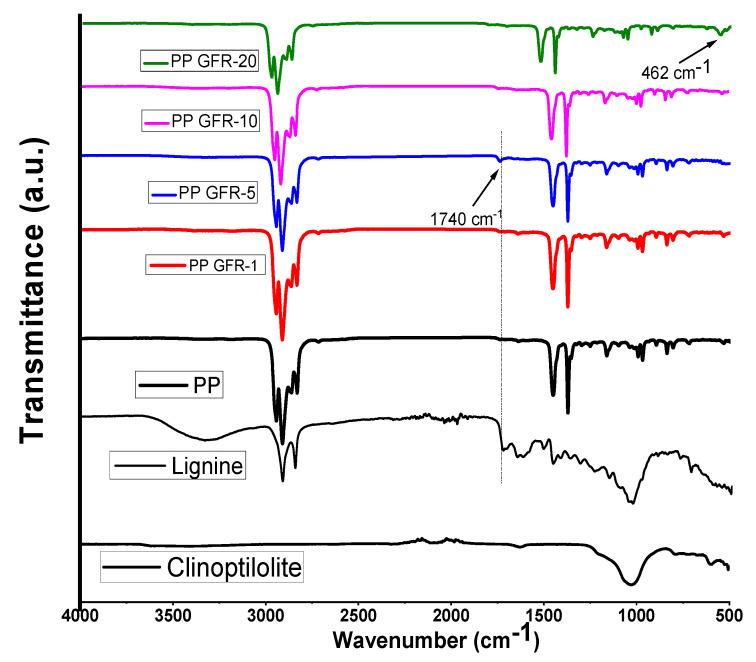
FTIR spectra of PP and PPGFR composites.

In Figure 2, the FTIR spectra of polypropylene and polymeric composites with zeolite and lignin are shown. The polypropylene spectrum showed signals at 2917 and 2840 cm^−1^ characteristics of the CH_2_-bond asymmetrical and symmetrical stretching [27]. At 1454 and 1369 cm^−1^, two intense signals assigned to the asymmetric bending of CH_2_ were located [28]; finally, in the PP spectrum, smaller signals were observed in the region from 1000 to 800 cm^−1^, which are attributed to CH_3_ oscillating vibration [29]. The PPGFR composites presented the same signals as pure PP; however, the PPGFR-5 sample presented a small signal at 1740 cm^−1^ attributed to the C=O bond belonging to lignin [30]. This weak signal was also detected in the PGFR-10 and PGFR-20 composites, and the dispersion and distribution of the particles was better in the PGFR-5 composite, which was why the signal was more intense. Only this signal belonging to lignin was detected, and a displacement of this signal was observed since it was originally located at 1730 cm^−1^. Several authors report that below the lignin content, no signals from the O-H groups are detected [31,32]. Abdelwahab et al. studied PP and lignin biocomposites and found the signal around 1740 cm^−1^ attributed to the carbonyl group [32]. Conversely, the PPGFR-20 spectrum presents a wide signal at 462 cm^−1^ corresponding to Si-O. In an earlier study of ours, this signal was only observed at high concentrations of clinoptilolite with Nylon 6 [33]. 

#### 3.1.2. X-ray Diffraction (XRD)

**Figure 3:**
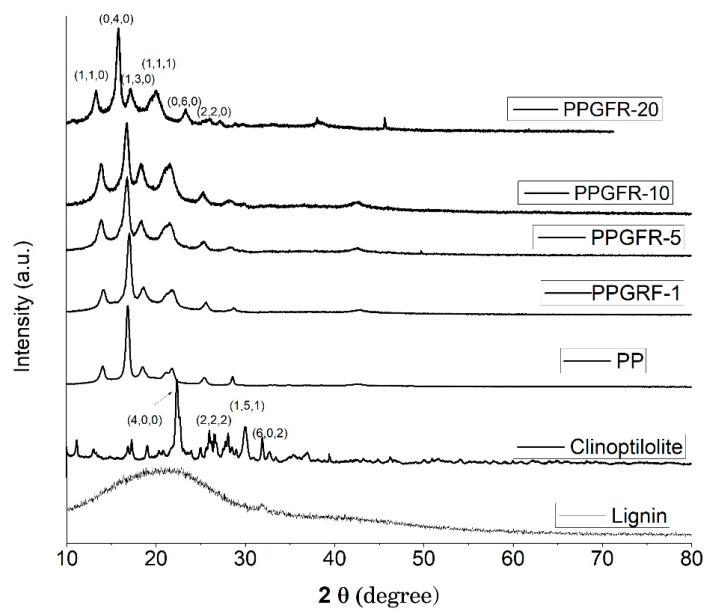
X-ray diffraction (XRD) patterns of the lignin, zeolite, PP, and PPGFR composites.

Figure 3 shows the X-ray diffraction (XRD) patterns of the lignin, zeolite, PP, and compounds obtained by the melt-extrusion process. Patterns identified in each of the composites obtained showed intense reflections at angles 13.76, 16.68, 18.22, 21.49, 25.25, and 28.19° corresponding to the Miller planes (110), (040), (130), (111), (060), and (220), respectively, of the crystalline phase of the PP [24]. 

The XRD pattern of pure lignin had a broad peak at around 21.5°, indicating its natural amorphous character [34]. Clinoptilolite exhibited peaks at 22.37, 26.63, 28.14, and 29.95°, consistent with the diffraction standard (JCPDS no. 39-1383), as well as being consistent with reported clinoptilolite XRD data [35].

The PPGFR-5 and PPGFR-10 composites showed a peak of very little intensity at 42.54° due to the presence of clinoptilolite. Conversely, in the PPGFR-20 composite, two peaks were observed at 38.16° and 45.56°. On the other hand, it was not possible to observe the presence of lignin in the composites due to its amorphous nature [24].

### 3.2. Thermal Stability Analysis

**Figure 4:**
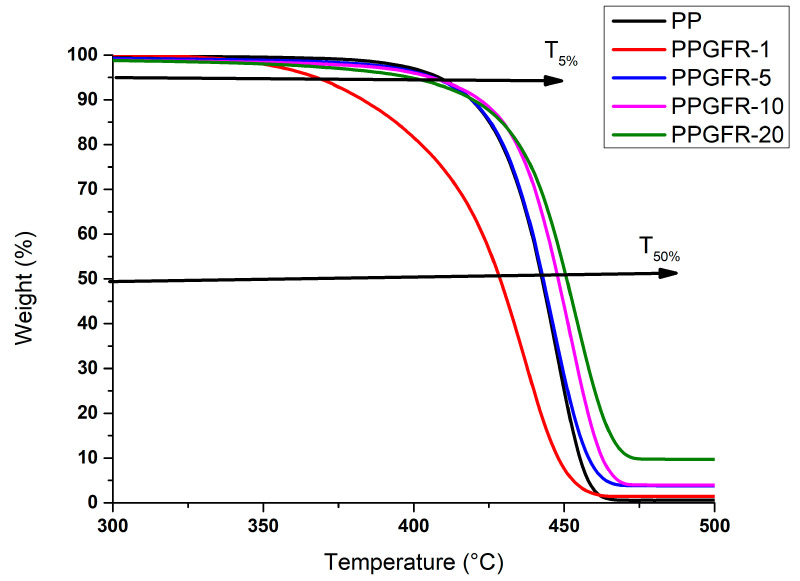
Thermogravimetric analysis of PP and composites obtained.

In Figure 4, the thermograms of PP polymer and polymeric composites obtained by melt extrusion are presented. For the polymer PP and PPGFR-5, PPGFR-10, and PPGFR-20 composites, a weight loss ranging from 393 °C to 470 °C can be appreciated. The PPGFR-20 sample presented the highest thermal stability; the weight loss in the region from 393 °C to 470 °C is attributed to the breakage of existing chains in the polypropylene structure [36]. 

The PPGFR-1 sample had a lower thermal stability than the other composites, even pure PP, as it showed a weight loss in the temperature range of 343 to 453 °C; this could have been due to the low lignin concentration and the fact that this material can melt easily. As reported by Shen et al. [37], the degradation process of Klason lignin could be divided into three stages of mass loss, which are (1) evaporation of moisture, (2) intensive evolution of aromatic compounds, and (3) formation of charcoal. Conversely, Brebu et al. [38] investigated that the temperature of the maximum mass loss rate varied between 350 and 400 °C using different lignin sources.

The temperature of the weight loss at 50% (T_50%_) of each composite and PP, as well as the amount of residue at 550 °C, are shown in Table 2. Results of T_50%_ were analyzed and compared, and we can observe that the thermal stability was proportionate directly to the amount of green flame retardants in the composite, obtaining a maximum increase of 10 °C for composites compared with the pure PP. This evidence suggests the formation of a thermally stable material created from the reaction of the polymeric matrix and the green flame-retardant mixture [39]. 

The thermal behavior presented by PPGFR composites was different from that reported for the PP-zeolite composite, since as the zeolite content in the composite increased, the degradation temperature of the composite decreased, and evidently the zeolite did not produce any improvement in the thermal properties of the material [40] 

Other reports indicate that zeolite combined with ammonium polyphosphate (APP) and pentaerythritol (PER) produces an efficient formulation as a flame retardant applied to ethylene copolymers [37]. In this report, zeolites showed greater fire protection when acid groups (COOH) were generated within the system during thermal decomposition. Polar ethylene copolymers and lignin may favor acid reinforcement of the analyzed material, and lignin also generates hydrogen sulfide that contributes to increasing the acidity [39,41,42].

### 3.3. Tensile Properties

The tensile properties of polypropylene and polypropylene composite with lignin and clinoptilolite were studied, and the results are presented in Table 3. The tensile strength and elongation at break for pure PP were 25.1 MPa and 247%, respectively. Compared with the PPGFR composite containing different concentrations of lignin and clinoptilolite, the tensile strength and elongation at break were reduced. Increasing the additive load content may cause deterioration of the mechanical properties of the polymeric matrix, due to low compatibility with additives [24,43]. Fortunately, the reduction in tensile strength was less than 20%, so for industrial applications where high tensile strength is not required, this new compound may be an alternative. In recent years, the automotive industry has been required to produce vehicles that do not harm the environment and have flame-retardant properties. This material can be applied as a filler for car doors, or textile fibers can be manufactured from them for roofs and car floors. Various studies have reported low mechanical properties when lignin is used as a polypropylene filler [44,45,46].

The elongation at break decreases drastically, probably because the composite has reduced flexibility due to the additive loading restricts the flow of the PP in a molten state. Composites of PP and starch, as a flame retardant, presented a decrease in elongation at break, attributed to the low compatibility between the matrix and filler, in addition to the additive restricting the flow of PP during processing [47].

### 3.4. Surface Morphology

**Figure 5:**
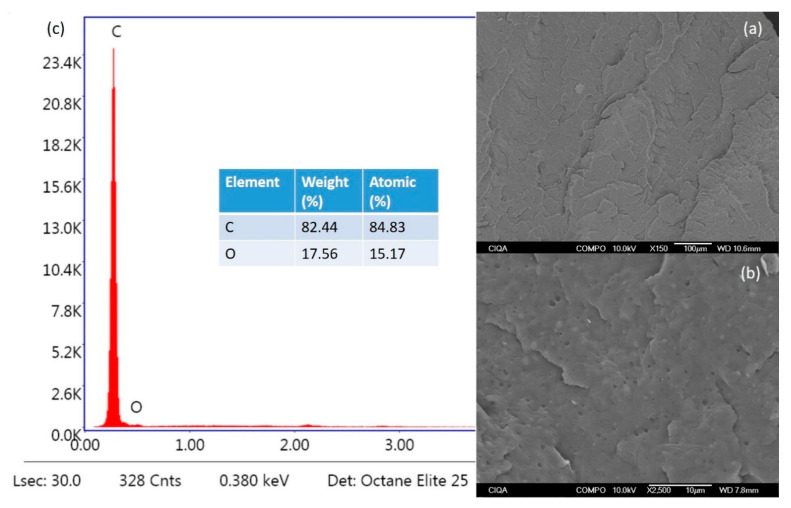
SEM images at different magnifications of pure polypropylene (**a**,**b**), and (**c**) EDS analysis.

Figure 5 shows SEM images of pure polypropylene at different magnifications (Figure 5a: 150 X and Figure 5b: 2500 X)) and the EDS analysis (Figure 5c). The morphological structure of the pure polypropylene surface shows that it was a smooth fracture surface containing no additives. EDS analysis shows only two elements: C (82.44 wt. %) and O (17.56 wt. %), characteristic of the chemical structure of polypropylene [24,48].

**Figure 6:**
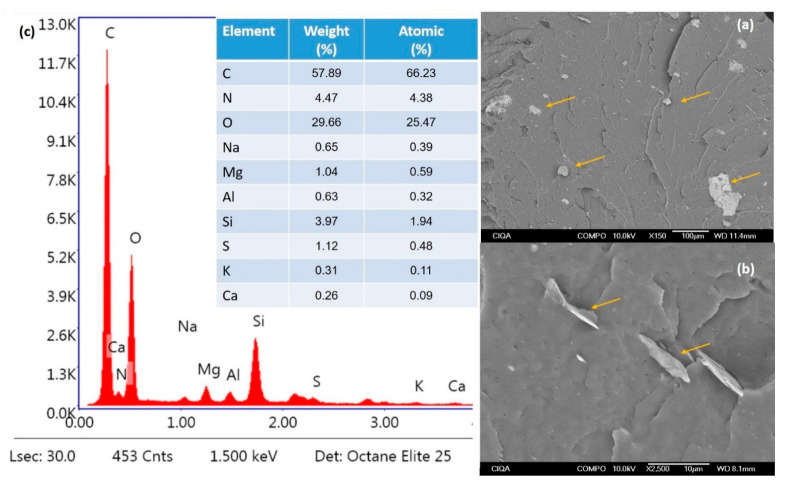
SEM images at different magnifications of PPGFR-20 ((**a**): 150X, (**b**): 2500X); (**c**) EDS analysis.

The SEM and EDS analysis of the PPGFR-20 composite presented evidence on the composition and distribution of the additives. Figure 6 shows two SEM images at different magnifications (Figure 6a: 150X and Figure 6b: 2500X), showing scattered lignin and clinoptilotite agglomerates in the polymeric matrix. At 2500X, zeolite and lignin particles embedded in the polymeric matrix were observed, and no deep fractures were observed, suggesting poor dispersion of green flame retardants. Although the melt extrusion process favors the dispersion of fillers in the matrix, some agglomerates were observed. EDS analysis (Figure 6c) confirmed the presence of the characteristic elements of clinoptilolite such as Al and Si, which were found in our previous work when clinoptilolite was incorporated into a nylon polymeric matrix [33]. A similar study by Wang et al. reports the finding of the same elements in zeolite [49]. The presence of elements such as C, O, N, Na, and S in the analysis is due to the presence of lignin in the matrix; this has been reported by other authors [50,51].

### 3.5. Flame Retardance Analysis

**Figure 7:**
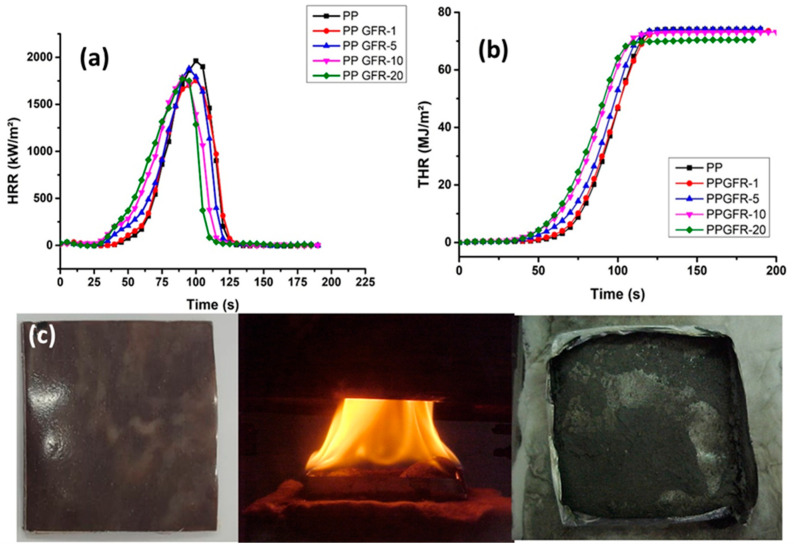
Images of cone calorimetry measurements of the PP and their composites. (**a**) Heat release rate, (**b**) total heat release, and (**c**) images of the residue after the flammability tests of PPGFR-20.

Figure 7 shows the heat release rate (HRR, Figure 7a)) and total heat release (THR, Figure 7b)) of PP and the polymer composites obtained. Table 4 shows the values of the cone calorimetric analysis and the residue obtained.

The HRR of PP was 1961.28 kW/m^2^, and the composites presented values of 1771.6 kW/m^2^ for PPGFR-1; 1881.51 kW/m^2^ for PPGFR-5; 1786.92 kW/m^2,^ and 1752.95 kW/m^2^ for PPGFR-10 and PPGFR-20, respectively (see Table 4). As can be seen, the HRR decreased with increasing concentration of the flame-retardant blend, decreasing up to 11.88% for PPGFR-20 compared to pure PP polymer. A parameter that allows for the evaluating the fire risk of a certain material is the HRR; in the PPGFR-20 sample, the peak was less intense, indicating that it exhibited a lower flame propagation and a lower amount of heat released. These results, together with the TGA data (Table 2), confirm that the combination of zeolite with lignin formed an effective green flame retardant for PP. Polymeric compounds have been reported where the zeolite confers properties that make the polymer more thermally stable, due to the formation of the intumescent layer that hinders combustion. This has been observed in zeolite-reinforced polyethylene copolymer, where the higher content of polar comonomer favors the anti-flame properties [39].

The increased thermal stability of the nylon/zeolite composite was demonstrated, since zeolite, due to its mesoporous structure, adsorbs heat, in addition to acting as a catalyst and stabilizing the residual carbon resulting from degradation [33]. Therefore, the existence of synergy between zeolite and another flame retardant such as lignin is suggested.

The THR (total heat release) values obtained are shown in Figure 7b, where for PP it was 70.18 MJ/m^2^, while for PPGFR-1, PPGFR-5, PPGFR -10, and PPGFR-20, samples were 71.62, 71.89, 70.78, and 67.87 MJ/m^2^, respectively (see Table 4), showing a decrease, in this parameter, as the flame retardant load increased. Zeolites combined with ammonium polyphosphate (APP) have been studied as additives for polypropylene flame retardants, and they demonstrated good flame-retardant properties, in addition to leaving higher carbonaceous residue [49]. The PPGFR composites presented a lower percentage of carbonaceous residues, which suggests that there is no effective reaction between the PP matrix and the lignin, allowing for the formation of thermally stable fragments.

A UL-94 test was performed to measure the flammability of the compounds, as well as the time required to extinguish combustion after the removal of the heat source. The rating obtained for PP and the PPGFR-1, PPGFR-5, and PPGFR-10 samples was V-2, which means that they have poor flame-retardant properties, while the PPGFR-20 compound obtained a V-0 and 5VB rating, which indicates that it has good flame-retardant properties. In this case, PPGFR-20 had no presented dripping of the material; therefore, the results agree with the percentage of residue and the reduction of HRR (see Table 4). The flame retardation index (FRI) was calculated with Equation (1) involving conical calorimetric data for thermoplastic composites. FRI < 1 is taken as the lowest level of flame retardation symbolized as “poor” performance [52,53]. According to the literature, a good flame retardation should have an FRI greater than 1; therefore, the PPGRF-20 composite is considered to have good antiflame, since it has a FRI of 1.15, which is in agreement with the UL-94 results [52,53]. Table 5 presents a comparison of several polymeric composites with green flame retardants classified by UL-94. All studies achieved V-0 classification.

### 3.6. Analysis of Char Residue

#### 3.6.1. Char Residue Surface Morphology

**Figure 8:**
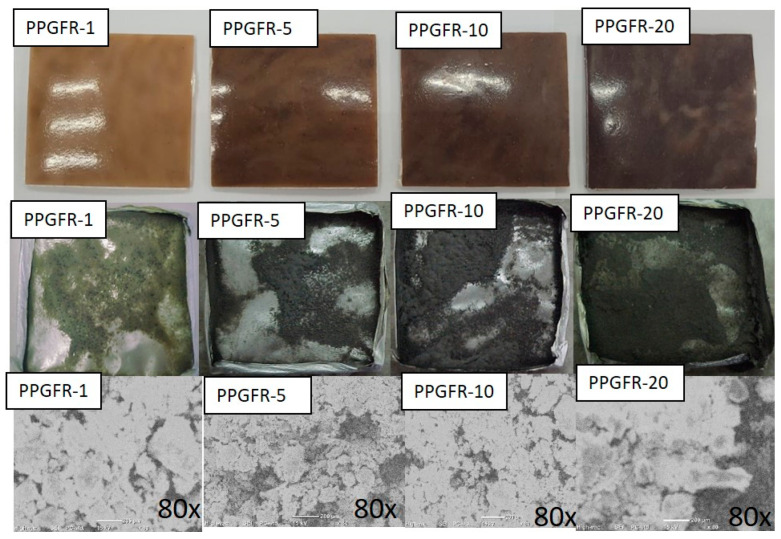
Digital photos and SEM images of composites PPGFR-1, PPGFR-5, PPGFR-10, and PP GFR-20.

Char residues were analyzed, and their morphology was thoroughly investigated after the conical calorimetric test. Figure 8 shows the images of char residues after the cone calorimetric test, where it is observed that the carbon layer increased as the percentage of green flame retardants increased. In the sample with 20% loading of green additives, a thick layer is observed, which coincided with the weight of the material reported in Table 4. In Figure 8, images PPGFR-5 and PPGFR-10 exhibit defects such as cracks and holes on the surface, which affect the flame retardancy, as heat, oxygen, and flammable gases penetrate through these cracks, causing the PP not to improve [58]. A thick or coarse carbon residue is mainly responsible for the flame-retardant properties [59]. 

Table 6 presents the results of the SEM-EDS analysis. The elements found were O, Al, and Si in all the composites; the element with the highest percentage by weight was Si, which is present in clinoptilolite. However, no elements belonging to the lignin structure were found, suggesting that there was no synergy between the two flame-retardant components (clinoptilolite and lignin) due to the presence of only the elements of the inorganic component.

#### 3.6.2. FTIR Spectroscopy

**Figure 9:**
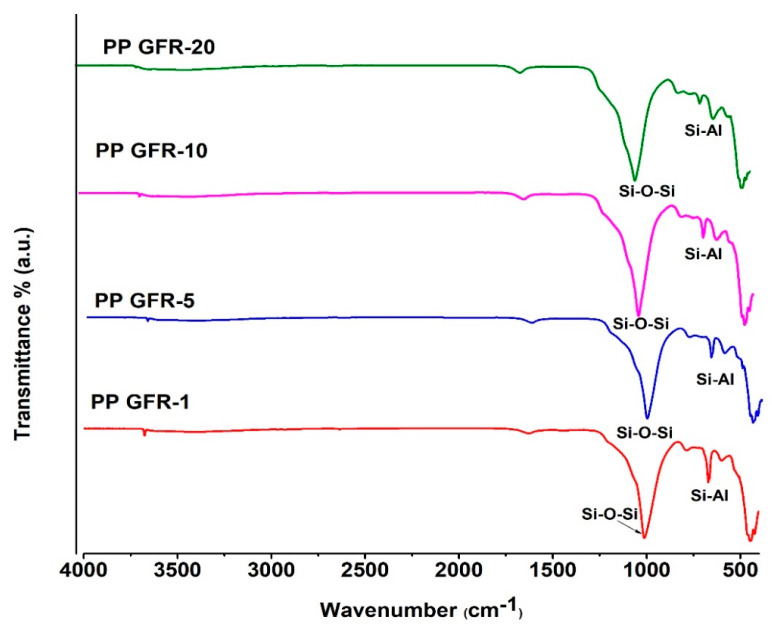
FTIR spectra char residue of PPGFR composites.

In all spectra (Figure 9), an intense signal was found at around 1019 cm^−1^ assigned to the Si-O-Si bond. The signal located at 670 cm^−1^ corresponds to the asymmetric stretching of the SI-Al tetrahedral atoms of the clinoptilolite structure [49,60]. SEM and FT-IR analyses agreed with the presence of inorganic compounds only. Both analyses indicate the formation of a carbon silicate rich in inorganic elements, which improves the barrier properties for heat and volatiles; the formation of this surface layer of carbon helps delay thermo-oxidative degradation [60,61]. It is known that the polyaromatic structure of lignin helps to reduce flame retardation, but in the PP/zeolite/lignin composite, this situation did not occur. It is also known that zeolite plays an important role in the formation of stable structures during the pyrolysis process. The TGA data in Table 2 indicate an increase in the thermal stability of PP to be combined with zeolite/lignin and suggest some interaction between the PP matrix and the green flame retardants, that is to say, the formation of a carbonized layer is possible. For this study, there was a moderate synergy between zeolite and lignin; this could be seen in the cone calorimetry tests, and it was considered that the char layer may have been more stable with the addition of some coupling agent.

#### 3.6.3. Flame-Retardant Mechanism

**Figure 10:**
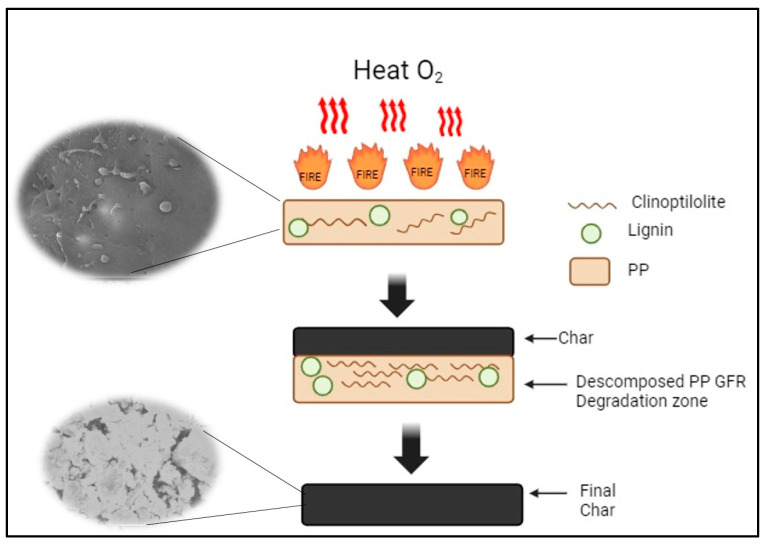
Mechanism of flame retardant by PPGFR composites.

Figure 10 shows the possible flame-retardant mechanism of PPGFR composites. It is known that PP is a thermoplastic with low thermal stability and is flammable; when it is exposed to fire, it does not generate any residue and is completely consumed. The opposite occurs with PP loaded with green flame retardants—the PPGFR composites with different concentrations of green flame retardants generate a carbon layer as residues. Due to the pyrolysis of the clinoptilolite, lignin is not part of this layer, according to the data obtained in the elemental analysis, indicating that it is consumed together with PP. Clinoptilolite acts as a carbon-silicate-forming agent rich in inorganic elements that provide protection against mass and heat transfer during the combustion process [62]. At a temperature of 450 °C, the aluminum oxide present in the clinoptilolite structure can release an amount of water, which can cool the polymer matrix, delaying its combustion and diluting the volatile gases [63]. Zeolite has acidic properties that promote catalytic activities; the structure and Si/Al ratio influence the acid conditions and the resistance of the zeolite. Heat treatment influences the formation of these acid sites; therefore, it is proposed that inorganic species being active catalysts leads to the formation of a carbon protector, and this was observed in the characterization by FT-IR and EDS analysis [64].

## 4. Conclusions

In conclusion, it was possible to obtain PP composites with two green flame retardants, lignin and clinoptilolite, both environmentally friendly and abundant in nature, as well as not being present any treatment before their incorporation into the polymer matrix. The melt extrusion method used for the manufacture of the PPFRG composites makes it an economical alternative, as it has several advantages such as its absence of solvents, being easily scalable to the industrial level, not generating by-products, and it being performed in a single step. PPGRF-20 composite is considered a flame-retardant material since it presented an FRI value of 1.15 and UL-94 V-0 and 5VB rating, and also showed a decrease in peak HRR of 11.88% compared to pure PP. The residue (char) showed cracks, and its elemental analysis indicated the presence of the elements Al, O, and Si, which was corroborate by FTIR. All PPGFR composites showed good dispersion and few aggregates. However, the tensile strength decreased as the weight percentage of lignin/clinoptilolite in the polymer matrix increased. For example, when only 1 wt% of lignin/clinoptilolite was incorporated, the tensile strength was 24.4 MPa, while when loading 20% of GRF blend, the tensile strength was 20.8 MPa.

Finally, it is concluded that lignin acted as a carbon source and not as a flame retardant; therefore, there was no synergy between the two green flame retardants.

## Figures and Tables

**Table 1:** The raw material composition of different PPGFRs.

Sample	PP (g)	Mix Green Flame Retardant (g)	Weight of Additive Clinoptilolite (g)	Weight of Additive Lignin (g)	Total Weight (g)
PP	300	0	0	0	300
PPGFR-1	297	3	2	1	300
PPGFR-5	285	15	10	5	300
PPGFR-10	270	30	20	10	300
PPGFR-20	240	60	40	20	300

(g) = grams.

**Table 2:** Results of the TGA analysis of PP and its composites.

Sample	Temperature at 5% of Weight Loss (T_5%_) (°C)	Temperature at 50% of Weight Loss (T_50%_) (°C)	Residue at 500 °C (%)
PP	410	441	0
PPGFR-1	368.	428	1.3
PPGFR-5	409	442	3.9
PPGFR-10	408	447	5.6
PPGFR-20	402	451	9.7

**Table 3:** Mechanical properties of PP and PPGRF composites.

Samples	Elongation at Break (%)	Tensile Strength (MPa)
PP	247 ± 1.80	25.1 ± 2
PPGFR-1	150 ± 1.25	24.4 ± 0.8
PPGFR-5	117 ± 1.55	23.6 ± 5.7
PPGFR-10	70 ± 2.7	23.3 ± 0.3
PPGFR-20	76 ± 5.4	20.8 ± 0.4

**Table 4:** Data of the cone calorimetry test of the samples analyzed.

Sample	Peak HRR (kW/m^2^)	THR (MJ/m^2^)	Residue %	FRI	UL-94
PP	1961.28 ± 0.6	70.18 ± 2.3	0	------	V-2
PPGFR-1	1771.6 ± 0.4	71.62 ± 1.2	0.38 ± 0.1	0.80	V-2
PPGFR-5	1881.51 ± 1.3	71.89 ± 0.5	2.25 ± 0.2	0.75	V-2
PPGFR-10	1786.92 ± 0.6	70.78 ± 1.1	4.1 ± 0.1	0.66	V-2
PPGFR-20	1752.95 ± 1.0	67.87 ± 0.5	9.86 ± 0.5	1.15	V-0

**Table 5:** Polymeric composites with green flame retardants and their classification according to UL-94.

Materials with Green Fire Retardants	UL-94 Classification	Tensile Strength (MPa)	References
PP and 30% hydrotalcite (LDHs-C)	V-0	16 ± 0.2	[43]
Polyurethane + polyphenol-iron-phytic acid	V-0	0.08	[54]
PLA+ phytic acid (PA) and furfuryl amine (FA)	V-0	44.7 ± 1.8	[55]
Bisphenol-A-type epoxy resin Imidazole (IM), ethanol, and phytic acid	V-0	------------	[56]
PP+ nano-graphitic lamellas	V-0	31.3	[57]
PPGFR-20	V-0	20.8 ± 0.4	This study

**Table 6:** SEM-EDS data of the char of PPGFR composites.

Samples	Element Weight (%)		
	O	Al	Si
PPGFR-1	12.83	29.98	57.19
PPGFR-5	24.47	16.28	59.25
PPGFR-10	14.49	17.40	68.11
PPGFR-20	24.10	7.42	68.47

## Data Availability

The data presented in this study are available on request from the corresponding authors.
